# Toward more robust and reproducible diffusion kurtosis imaging

**DOI:** 10.1002/mrm.28730

**Published:** 2021-04-08

**Authors:** Rafael N. Henriques, Sune N. Jespersen, Derek K. Jones, Jelle Veraart

**Affiliations:** ^1^ Champalimaud Research Champalimaud Centre for the Unknown Lisbon Portugal; ^2^ Center of Functionally Integrative Neuroscience (CFIN) and MINDLab Department of Clinical Medicine Aarhus University Aarhus Denmark; ^3^ Department of Physics and Astronomy Aarhus University Aarhus Denmark; ^4^ CUBRIC School of Psychology Cardiff University Cardiff UK; ^5^ Mary MacKillop Institute for Health Research Australian Catholic University Melbourne Victoria Australia; ^6^ Center for Biomedical Imaging New York University Grossman School of Medicine New York NY USA

**Keywords:** diffusion MRI, DKI, kurtosis, parameter estimation, robustness

## Abstract

**Purpose:**

The general utility of diffusion kurtosis imaging (DKI) is challenged by its poor robustness to imaging artifacts and thermal noise that often lead to implausible kurtosis values.

**Theory and Methods:**

A robust scalar kurtosis index can be estimated from powder‐averaged diffusion‐weighted data. We introduce a novel DKI estimator that uses this scalar kurtosis index as a proxy for the mean kurtosis to regularize the fit.

**Results:**

The regularized DKI estimator improves the robustness and reproducibility of the kurtosis metrics and results in parameter maps with enhanced quality and contrast.

**Conclusion:**

Our novel DKI estimator promotes the wider use of DKI in clinical research and potentially diagnostics by improving the reproducibility and precision of DKI fitting and, as such, enabling enhanced visual, quantitative, and statistical analyses of DKI parameters.

## 1 INTRODUCTION

Despite a growing interest in biophysical models of diffusion in white matter to develop specific biomarkers of microstructural changes,^1^ signal representations, for example, diffusion tensor imaging (DTI)^2^ or diffusion kurtosis imaging (DKI),^3^ retain the potential to become invaluable tools in diagnostic and clinical research settings. Such signal representations provide metrics that are highly sensitive to microstructural changes associated with development,^4^, ^5^ aging ^6^, ^7^, disease and disorder without adopting (often contested^1^, ^8^, ^9^, ^10^) model assumptions. An extensive literature has demonstrated the sensitivity of DKI parameters to white and gray matter alterations in, for example, aging,^7^, ^11^, ^12^, stroke,^13^, ^14^, ^15^, ^16^, ^17^ traumatic brain injury,^18^, ^19^, ^20^ multiple sclerosis,^21^ schizophrenia,^22^, ^23^ autism spectrum disorder,^24^, ^25^ epilepsy,^26^, ^27^ migraine,^28^ Parkinson’s Disease,^29^ and Alzheimer’s disease.^30^, ^31^ Despite a focus on neuroapplications, DKI also has emerging applications in body MRI.^32^, ^33^ However, sensitivity is not enough; diagnostics and clinical research may only adopt biomarkers that are also accurate, precise, and robust.^34^

Technically, DKI forms a straightforward extension of DTI and provides, aside from the diffusion tensor, an estimate of the diffusion kurtosis tensor which quantifies the degree of directional non‐Gaussian diffusion.^3^, ^35^, ^36^ DKI parameters, for example, the mean kurtosis ($\bar{K}$), radial kurtosis ($K_{⊥}$), and axial kurtosis ($K_{\|}$),^35^, ^36^, ^37^ have been shown to yield clinically relevant information that is not captured by a more conventional DTI representation. Moreover, DTI parameters (eg, mean diffusivity, radial diffusivity, axial diffusivity and fractional anisotropy^38^) are themselves estimated more accurately when evaluated within the DKI framework (ie, estimating the diffusion tensor and kurtosis tensor simultaneously).^39^ Compared to DTI, DKI requires a slightly more extensive scan protocol in the sense that there must be at least 3 distinct *b*‐values (typically one of these is set to b=0), which differ only in their gradient magnitude.^3^, ^40^ Given the widespread availability of accelerated image acquisition techniques such as simultaneous multiband imaging,^41^, ^42^ data compatible with whole‐brain DKI‐analysis can be acquired in a few of minutes, thereby facilitating clinical utility. Moreover, many recent large cohort studies, for example, the Human Connectome Project,^43^, ^44^ UK Biobank,^45^ Adolescent Brain Cognitive Developement,^46^ and the Cambridge Centre for Ageing Neuroscience (CamCAN),^47^, ^48^ provide now large‐scale multishell data that are well‐suited to DKI analyses.

Unfortunately, DKI has been challenged ever since its introduction by a poor robustness to imaging artifacts and thermal noise that often leads to non‐physical kurtosis values during the fitting, especially in voxels with very low radial diffusivity.^37^, ^49^, ^50^, ^51^ Indeed, implausible negative values are ubiquitous in many kurtosis maps, hampering visual, quantitative, and statistical analysis of the data. Advances in artifact correction, noise removal, and constrained parameter estimation have reduced, but not eliminated the problem.^37^, ^49^, ^52^, ^53^, ^54^, ^55^ Alternatively to the full tensor estimations, a scalar kurtosis quantity can be obtained from diffusion‐weighted signals averaged across different isotropically distributed gradient directions (and for each individual *b*‐value): that is, the powder kurtosis $\dot{K}$,^10^, ^12^, ^56^ distinct from mean kurtosis. Due to the higher signal‐to‐noise ratio (SNR) of the powder signals and decreased number of estimated model parameters, $\dot{K}$ can be computed with higher precision than other kurtosis tensor derived metrics. We will demonstrate in this work that $\dot{K}$ can be used to provide a robust prediction of the mean kurtosis without the need to estimate the kurtosis tensor. However, the powder‐averaged signal does not allow for the estimation of directional kurtosis values, which have been shown to provide unique information in well‐aligned structures.^57^, ^58^, ^59^ Moreover, a robust estimation of the full kurtosis tensor is required for biophysical modeling^60^, ^61^, ^62^ and tractography.^63^, ^64^, ^65^

Here we propose and evaluate a novel technique for more robust and precise estimation of the full kurtosis tensor and derived metrics. We introduce a regularized DKI estimator in which the estimated mean kurtosis is evaluated against a robust prediction of the mean kurtosis, which in turn is derived from the powder kurtosis. We will describe the technical details and demonstrate how they improve the reproducibility, robustness and precision of DKI parameters.

## 2 METHODS

### 2.1 Diffusion kurtosis imaging

DKI provides a representation of the diffusion‐weighted signal *S* in terms of the second and fourth cumulant as a function of the diffusion‐weighting strength,^3^ or b‐value, *b*. In 1D or in the case of isotropic diffusion,

(1) $$\begin{array}{c} {\log S\left(b\right)=S|}_{b=0}‐bD_{\mathrm{APP}}+\frac{1}{6}b^{2}D_{\mathrm{APP}}^{2}K_{\mathrm{APP}}+O\left(b^{3}\right), \end{array}$$

with $D_{\mathrm{APP}}$ and $K_{\mathrm{APP}}$ the apparent diffusion and apparent kurtosis coefficients, respectively. Since proton diffusion in biological tissue, e.g. brain white matter, is typically anisotropic, 3D diffusion and kurtosis tensors are needed to describe the orientational dependence of the diffusion‐weighted signal adequately^40^, ^65^:

(2) $$\begin{array}{cc} \log S(b,g) & =S{|}_{b=0}‐b\sum_{i,j=1}^{3}g_{i}g_{j}D_{\mathrm{ij}}+\frac{1}{6}b^{2}{\bar{D}}^{2}\sum_{i,j,k,l=1}^{3}\;g_{i}g_{j}g_{k}g_{l}W_{\mathrm{ijkl}}+O(b^{3})\\ & =S{|}_{b=0}‐bD_{\mathrm{APP}}(g)+\frac{1}{6}b^{2}D_{\mathrm{APP}}{\left(g\right)}^{2}K_{\mathrm{APP}}(g). \end{array}$$

Here, g is the unit vector along which the diffusion‐weighting gradient is applied, and $D_{\mathrm{ij}}$ represents the $ij^{th}$ element of the fully symmetric second‐order diffusion tensor D for which a third of the trace equals the mean diffusivity $\bar{D}$. In addition, $W_{\mathrm{ijkl}}$ denotes the ${\mathrm{ijkl}}^{th}$ element of the fully symmetric fourth order diffusion kurtosis tensor W. Because both tensors are fully symmetric, D and W have 6 and 15 degrees of freedom, respectively.

From D and W, various diffusion and kurtosis parameters can be derived.^35^, ^37^, ^38^, ^66^, ^67^ Fractional anisotropy (FA), mean diffusivity ($\bar{D}$), radial diffusivity ($D_{⊥}$), axial diffusivity ($D_{\|}$), mean kurtosis ($\bar{K}$), radial kurtosis ($K_{⊥}$), and axial kurtosis ($K_{\|}$) are amongst the most widely adopted DKI parameters.^35^, ^36^, ^62^, ^65^ Without loss of generality we here limit ourselves to the original definition of mean kurtosis $\bar{K}$, computed by evaluating the apparent kurtosis from the $K_{\mathrm{APP}}\left(g\right)$ along a large number of directions g followed by averaging.

### 2.2 Powder kurtosis

Analogous to Equation (1), the decay of the powder‐averaged signals $\dot{S}$ as a function of *b* can be approximated using the cumulant expansion^10^, ^12^:

(3) $$\begin{array}{c} \log\dot{S}(b)=S{|}_{b=0}‐b\dot{D}+\frac{1}{6}b^{2}{\dot{D}}^{2}\dot{K}+O(b^{3}), \end{array}$$

where $\dot{D}$ and $\dot{K}$ are the scalar diffusivity and excess‐kurtosis of powder signals, respectively. Since powder‐averaged signals are independent of the orientation distribution of microscopic components,^56^, ^68^, ^69^, ^70^ the scalar quantity $\dot{K}$ extracted from these signals are decoupled from mesoscopic properties such as tissue dispersion or fiber architecture configurations.

### 2.3 Robust prediction of mean kurtosis

In this section, we will present 3 strategies to predict the mean kurtosis $\hat{K}$ without relying on the full tensor. First, from the powder kurtosis, $\hat{K}$ can be computed analytically^10^ from the signal in the limit b→0:

(4) $$\begin{array}{c} {\hat{K}}^{\left(1\right)}=\dot{K}‐\Psi , \end{array}$$

with

(5) $$\begin{array}{c} \Psi =\frac{2}{5}\frac{D_{11}^{2}+D_{22}^{2}+D_{33}^{2}+2D_{12}^{2}+2D_{13}^{2}+2D_{23}^{2}}{{\bar{D}}^{2}}‐\frac{6}{5}. \end{array}$$

The correction term ψ mainly depends on the anisotropy captured by the elements of the diffusion tensor $D_{\mathrm{ij}}$. Importantly, the derivation of this expression relies on the mean kurtosis tensor definition proposed by Hansen et al,^36^, ^67^ which was shown to present nearly identical contrast to the original mean kurtosis definition $\bar{K}$.^36^

Second, for an isotropic diffusion tensor D, the correction term ψ is zero and $\dot{K}$ yields an accurate prediction of the mean kurtosis:

(6) $$\begin{array}{c} {\hat{K}}^{\left(2\right)}=\dot{K}. \end{array}$$

Third, for finite *b*‐values, a mean kurtosis prediction can be obtained from $\dot{K}$ and the diffusion tensor D using a polynomial regression model in which the thousands of non‐problematic voxels, that is, positive apparent kurtosis in each direction, serve as training data. Note that due to the strong dependence of DKI parameters on scan settings, *b*‐values,^71^ and subject‐specific alteration of the underlying tissue microstructure, the training voxels must be sampled from the same subject or from datasets with the same acquisition parameters. In this strategy, coined *voxel quality transfer*, the polynomial coefficients $C_{N}$ are estimated using multivariate polynomial regression in which the parameters $\bar{D}$, $\dot{K}$, and $\delta =D_{11}^{2}+D_{22}^{2}+D_{33}^{2}+2D_{12}^{2}+2D_{13}^{2}+2D_{23}^{2}$ are included up to the $N^{\mathrm{th}}$ order. Hence,

(7) $$\begin{array}{c} {\hat{K}}^{\left(3\right)}=P_{N}\left(\dot{K},\bar{D},\delta |C_{N}\right) \end{array}$$

with $P_{N}$ a multivariate polynomial function of order *N*. In this work N=3.

To summarize, the predicted mean kurtosis $\hat{K}$ can be estimated robustly from the diffusion tensor D and the powder kurtosis $\dot{K}$ using 3 strategies: (a) powder kurtosis with analytical correction, (b) without analytical correction, or (c) voxel quality transfer using polynomial regression. Where deemed relevant, the strategy will be specified using the superscript index. If not specified, $\hat{K}\equiv {\hat{K}}^{\left(3\right)}$ as motivated by our results (see below).

### 2.4 Regularized kurtosis fitting

After establishing a robust prediction of the mean kurtosis $\hat{K}$ (using 1 of the 3 strategies described above), we will use this metric to regularize the DKI fitting. Here we propose the following regularized nonlinear least squares (NLS) estimator:

(8) $$\begin{array}{c} \hat{\theta }={\text{arg min}}_{\theta }{\left\|S‐\text{exp}\left(‐B\theta \right)\right\|}^{2}+\alpha {\left\|h\left(\theta \right)‐\hat{K}\right\|}^{2}, \end{array}$$

with α the regularization weight and *h* the operator that computes the mean kurtosis $\bar{K}$ from the tensor coefficients θ.^36^, ^67^ Note that exp(‐Bθ) is an alternative formulation of the DKI model in Equation (2) in which *B* represent the extended *b*‐matrix.^72^ For α=0, the estimator reduces to the ordinary NLS estimator, a widely adopted estimator for DTI and DKI.^37^, ^49^, ^73^

We hypothesize that the prediction of the mean kurtosis $\hat{K}$ from the powder‐averaged data is a much more robust and reproducible metric than $\bar{K}$ estimated from the fitted kurtosis tensor and, as such, the $L_{2}$ norm of the difference between $\bar{K}$ and $\hat{K}$ regularizes and stabilizes the DKI fit.

The nonlinear fitting is initiated by a starting point obtained by fitting the DKI model using the ordinary NLS estimator. In the few cases where the estimator does not converge to a plausible solution, the estimation is repeated with a starting point that is the result of a constrained DKI fitting in which positivity of $K_{\mathrm{APP}}\left(g\right)$ is imposed.

### 2.5 Alternative fitting strategies

We compare performance of the regularized NLS estimator to commonly used fitting strategies: (a) ordinary NLS, (b) ordinary NLS after smoothing the data with a 2D isotropic Gaussian filter with a [5×5] kernel and full‐width‐half‐maximum of 1.25 times the voxel size, and (c) constrained NLS. The constrained NLS estimator solves the same objective function as the ordinary NLS estimator, but imposes one or more constraints on the estimated parameters.^37^ Here, we only adopted the constraint that the apparent kurtosis coefficient $K_{\mathrm{APP}}\left(g\right)$ must be positive in each gradient direction g.

### 2.6 Data

#### 2.6.1 Simulated data

Monte Carlo simulations with 2500 trials were performed to evaluate the performance of the regularized DKI estimator. For each trial, diffusion‐weighted signals were generated by evaluating the DKI signal for the diffusion encoding scheme of our study‐specific data (vide infra). The ground truth diffusion tensor and diffusion kurtosis tensor coefficients were randomly sampled from the plausible DKI estimates of the HCP data. Gaussian noise was added to the simulated data with a corresponding SNR of 30 for the non‐diffusion‐weighted signal. We opted for Gaussian noise instead of complex Gaussian noise to avoid the Rician noise bias as a confound in our interpretation.^74^ The effect of Rician bias on the estimation of DKI parameters has previously been studied and documented in detail.^49^, ^72^, ^75^

#### 2.6.2 Study‐specific MRI data

Data were collected under the approval of the Cardiff University School of Psychology Ethics Committee. Five healthy volunteers were recruited and data were collected on 2 different scanning sessions with exactly the same imaging protocol on a Siemens Connectom 3T MR scanner using a 32‐channel receiver coil. For each volunteer, the 2 test‐retest scanning sessions were performed on the same day interleaved by a short break. In both sessions, subjects were re‐positioned by the same operator. The repeated images were analyzed individually, without data averaging.

The diffusion gradients were characterized by Δ/δ=30/15ms and maximal gradient amplitude of 78mT/m; note that in this work the full power of the Connectom gradient system was not exploited to ensure translation of the findings to clinical scanners (where gradient amplitudes of 80 mT/m are prevalent). Diffusion weighting was applied along 30 isotropically‐distributed gradient directions^76^ for b= 0.5, 1, and 2.5 $\;\mathrm{ms}/\mu m^{2}$, with $\mathrm{TR}/T_{E}:3500/66\;\mathrm{ms}$, matrix: 88×88, and 54 slices with a spatial resolution of $2.5\;\times \;2.5\times \;2.5\;{\mathrm{mm}}^{3}$. Data acquisition was accelerated using simultaneous multiband (SMS = 2) and GRAPPA (R = 2), but partial Fourier encoding was turned off. In addition, 4 non‐diffusion‐weighted images were acquired with the same and reversed phase encoding to enable susceptibility‐induced geometrical correction. The total acquisition time was 6 m 15 seconds per session.

#### 2.6.3 Additional MRI data

Additional experiments are based on arbitrarily selected data sets from various public neuroimaging repositories (eg, Openneuro) that include multi‐shell diffusion‐weighted MRI data. We selected data from a wide variety of (clinical) research projects: MGH Adult Diffusion Data from the Human Connectome Project (HCP,^43^, ^44^, ^77^), the MASiVar project,^78^ the Mexican substance use disorder database neuroimaging dataset (SUDMEX),^79^, ^80^, ^81^ and Cambridge Centre for Ageing Neuroscience (CamCAN) dataset inventory.^47^, ^48^ Although all datasets meet the minimal requirements for DKI analysis, various scan and subject parameters vary widely. A comprehensive summary of the protocols and subjects is presented in Table 1—full protocol details can be found on each project’s resource pages.

### 2.7 Image processing

The study‐specific data were corrected for Gibbs ringing,^82^ eddy current and susceptibility‐induced geometrical distortions,^83^, and gradient nonlinearities^84^ prior to DKI analyses. As a part of the eddy current correction, signal outlier detection to identify, for example, motion‐corrupted slices was performed.^85^ Seven regions of interest (ROIs) were automatically segmented in the native spaces of the images to minimize data interpolation. The ROIs included major white matter tracts, that is, genu and splenium of the corpus callosum (GCC and SCC), corticospinal tract (CST), arcuate fasciculus (AF), inferior fronto‐occipital fasciculus (IFO), superior longitudinal fasciculus (SLF), and optic radiation (OR). The automated segmentation of all white matter bundles was performed using the TractSeg algorithm.^86^

**TABLE 1:** Overview of the scan parameters

	HCP	MASiVar	SUDMEX	CamCAN	Study‐specific data
**Scan protocol**					
$T_{E}$ (ms)	57	79	127	104	66
*b*‐values $\left(\mathrm{ms}/\mu m^{2}\right)$	0, 1, 3	0, 1, 2	0, 1, 3	0, 1, 2	0, 0.5, 1, 2.5
# directions	5, 64, 64	16, 40, 56	8, 32, 96	3, 30, 30	5, 30, 30, 30
Voxel size $\left({\mathrm{mm}}^{3}\right)$	1.25×1.25×1.25	2.1×2.1×2.2	2×2×2	2×2×2	2.5×2.5×2.5
**Subject information**					
Age (years)	40‐44	23	43	66	26
Gender	F	M	M	M	F
**Preprocessing**					
	HCP^77^	PreQual^87^	Designer^52^	Designer^52^	see Section 2.6.2

If (minimally) preprocessed data were available for the cohort data, then we used those data sets without applying additional processing steps for DKI analyses. Otherwise, the DESIGNER pipeline was applied to correct for thermal noise, Gibbs ringing, and eddy current distortions.^52^ This information is also listed in Table 1.

### 2.8 Statistics

The test‐retest reliability of the estimated ROI‐averaged $\bar{K}$ was evaluated for each fitting strategy using the test‐retest variability (TRV). Note that the test and retest data were not aligned to each other, since reproducibility was only evaluated for ROIs that were segmented in the native spaces of the images to minimize data interpolation. The TRV was computed across *N* subjects by averaging the ratio of the absolute difference and the average of the test‐retest estimates over the N=5 subjects. A scaling factor of $\sqrt{\pi }/2$ was applied to derive the standard deviation from the mean absolute difference for Gaussian distributed variables.

## 3 RESULTS

### 3.1 Robust prediction of mean kurtosis

**FIGURE 1:**
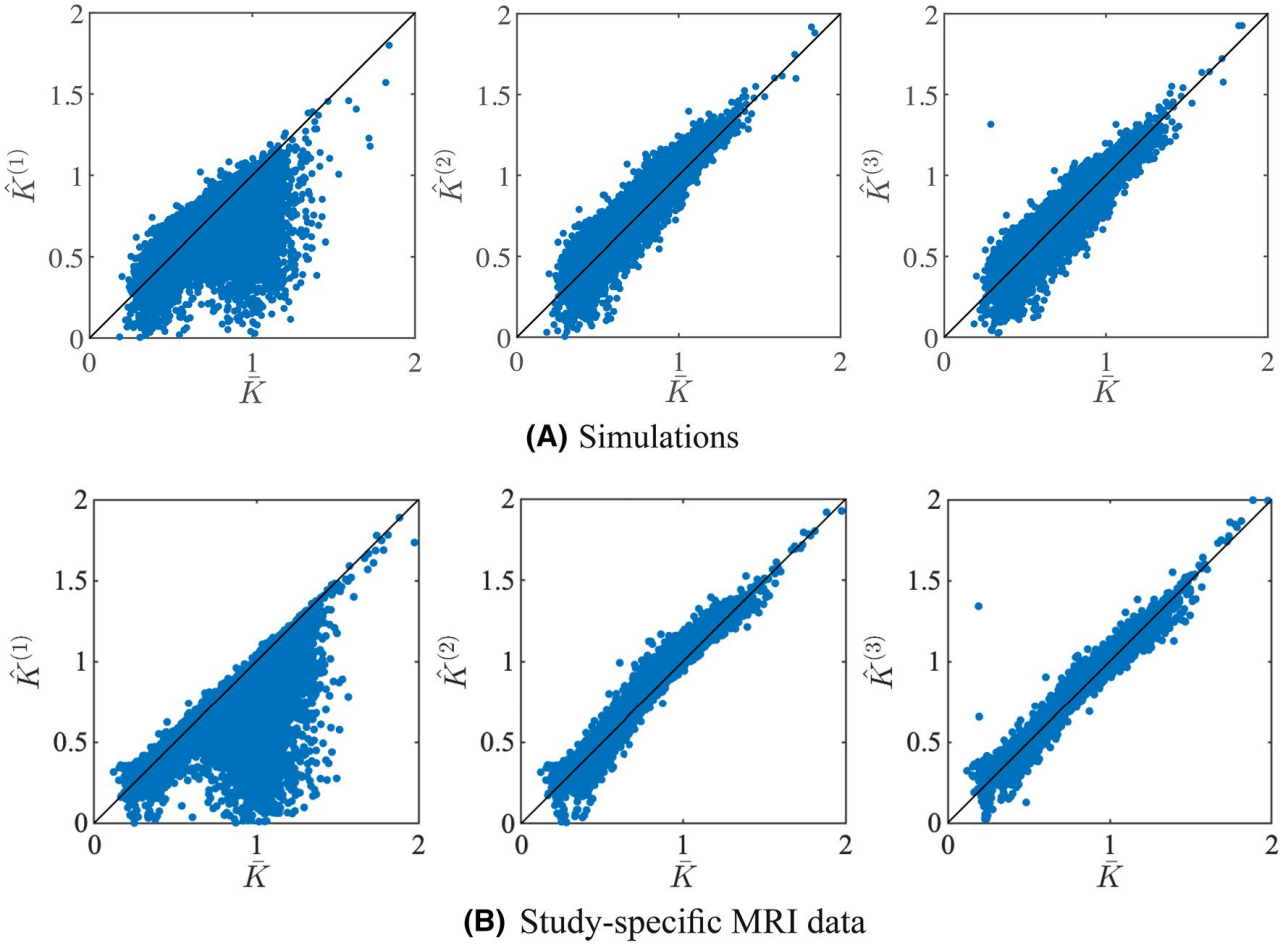
Simulations: a scatter plot shows the relationship between the actual mean and predicted mean kurtosis for the simulated and study‐specific data

In Figure 1, we show the scatter plot of predicted mean kurtosis $\hat{K}$ against the corresponding $\bar{K}$ in simulations and experimental data, using the 3 prediction strategies. In the simulations, $\bar{K}$ is the ground truth value, whereas for the experimental data $\bar{K}$ is estimated using an ordinary NLS estimator. For the experimental data, we only include all 441,271 voxels of the 5 study‐specific *retest* data with positive $\bar{K}$. The similarity between $\bar{K}$ and $\dot{K}$ (or ${\hat{K}}^{\left(2\right)}$) is high. The voxelwise percentage error is 3.4±11.50% and 1.64±5.26% (mean ± standard deviation) for the simulations and all experimental data, respectively. The correction using the analytical correction term Ψ (${\hat{K}}^{\left(1\right)}$) reduces the accuracy significantly, leading to an increase of the mean percentage errors with an order of magnitude; ‐10.24±17.56% and ‐7.63±12.02% for the simulations and all experimental data, respectively. The third strategy, that is, correction using polynomial dictionary learning (${\hat{K}}^{\left(3\right)}$), provides more accurate predictions of mean kurtosis than the other two. The mean percentage error is only 0.05±11.08% for the simulated data and 0.10±5.21% for the experimental data. Of importance, the polynomial regression model was trained on all voxels of the 5 study‐specific *test* data with positive $\bar{K}$. The Pearson correlation coefficients between $\bar{K}$ and ${\hat{K}}^{\left(1\right)}$, ${\hat{K}}^{\left(2\right)}$, and ${\hat{K}}^{\left(3\right)}$ was 0.70, 0.95, and 0.95, respectively, for the simulated data, and 0.82, 0.99, and 0.99, respectively, for the experimental data.

**FIGURE 2:**
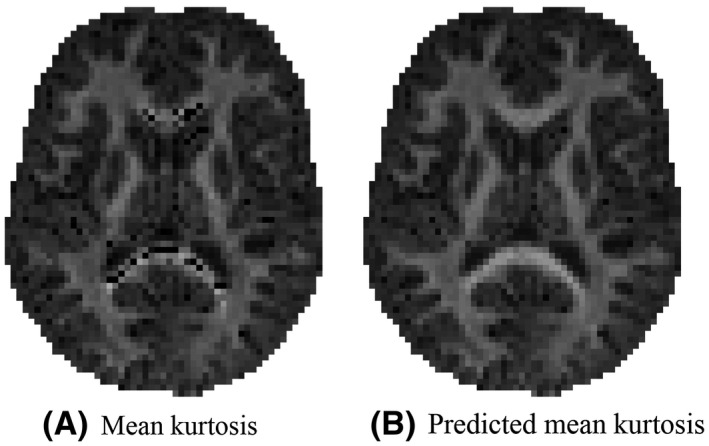
An representative map of the mean kurtosis $\bar{K}$ and the predicted mean kurtosis $\hat{K}$‐as estimated using the third strategy: voxel quality transfer using polynomial regression. The grayscale intensities are scaled between 0 and 2

In Figure 2, it is apparent that the predicted mean kurtosis ${\hat{K}}^{\left(3\right)}$ is more robust than $\bar{K}$. Indeed, the prevalence of the ubiquitous “black voxels” in major white matter structures is reduced significantly, even almost nullified. The percentage of voxels in the whole brain with negative $\bar{K}$ varies from 0.95 to 3.27%—with an average of 1.84%—in the ten study‐specific data sets. In contrast, for ${\hat{K}}^{\left(3\right)}$, this percentage dropped on average to 0.07%. Therefore, the quality of the maps is increased, allowing for a less confounded visual and statistical analysis. The robustness is similar for all 3 strategies to predict the mean kurtosis.

Based upon the above results, we opt for the third strategy to compute the predicted mean kurtosis $\hat{K}$. Hence, for the remainder of this work, $\hat{K}\equiv {\hat{K}}^{\left(3\right)}$.

### 3.2 Accuracy of regularized tensor fitting

**FIGURE 3:**
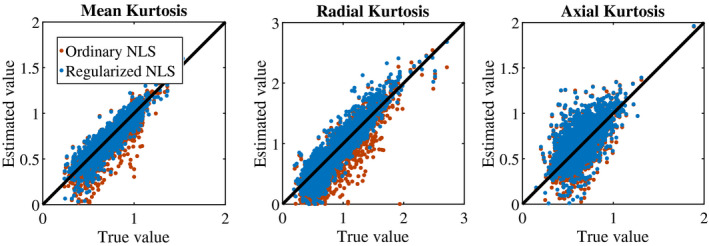
Simulations: Scatter plots show the relationship between the ground truth (“reference") and estimates of mean kurtosis ($\bar{K}$), radial kurtosis ($K_{⊥}$), and axial kurtosis ($K_{\|}$) when fitting the DKI model using the ordinary (red) and regularized (blue) nonlinear least squares estimator

Figure 3 summarizes the simulation results by showing the ground truth reference of DKI parameters versus their estimates obtained using the ordinary (red) and regularized NLS (blue) estimator. The average percentage difference between the estimated and ground truth mean kurtosis is ‐4.14±14.12 and ‐0.65±11.30% for the ordinary and regularized NLS estimators. For the radial kurtosis, the average percentage difference decreases from ‐11.14±28.53 to ‐5.10±21.72% when regularizing the tensor fitting. For axial kurtosis, the percentage difference is minimally altered: 5.20±28.30 and 6.07±28.35%.

**FIGURE 4:**
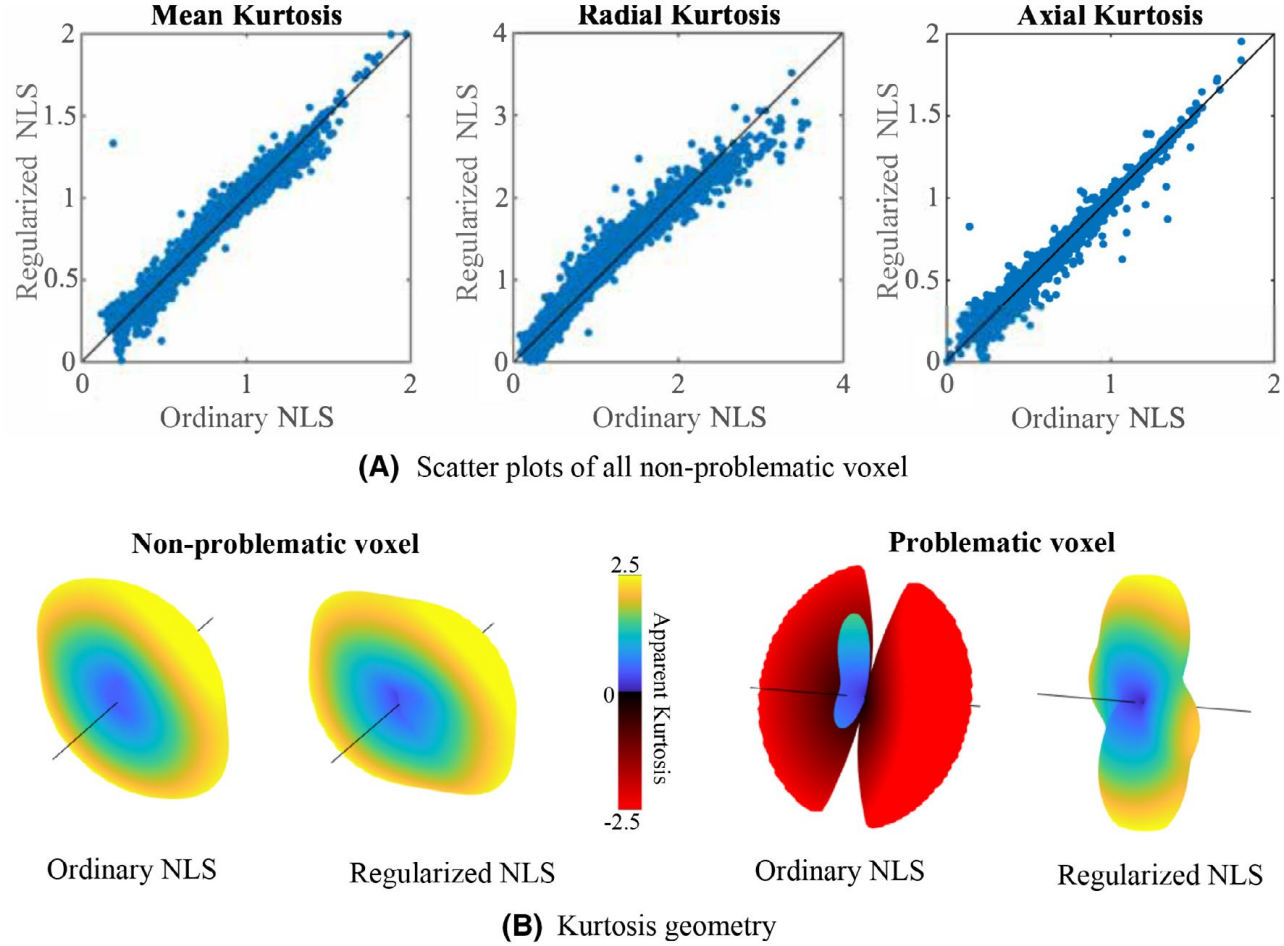
Study‐specific MRI data: A, Scatter plots show the relationship between the ordinary and regularized NLS estimates of mean kurtosis ($\bar{K}$), radial kurtosis ($K_{⊥}$), and axial kurtosis ($K_{\|}$) for all non‐problematic voxels in the study‐specific MRI data. Note that a “problematic voxel” has at least one negative $K_{\mathrm{APP}}$ when fitted with the ordinary NLS. B, The 3D kurtosis geometry, ie, apparent kurtosis coefficient evaluated along different directions, as estimated with the ordinary and regularized NLS for a non‐problematic and problematic voxel. These 3D kurtosis geometry were extracted from a voxels near the mid‐sagittal plane of the genus of the corpus callosum (direction marked by the black line corresponds to the principal direction of the diffusion tensor and $K_{\mathrm{APP}}\leq ‐2.5$ are truncated

For the study‐specific data, the ground truth kurtosis values are missing. However, for the majority of the gray and white matter voxels, the ordinary NLS estimator yields plausible and non‐problematic estimates with positive kurtosis values. In Figure 4A, we show the correspondence between estimated kurtosis parameters using the ordinary and regularized NLS estimator for those voxels. The average percentage difference between the ordinary and regularized NLS estimators for $\bar{K}$, $K_{⊥}$, and $K_{\|}$ is ‐0.15±3.87%, ‐0.71±6.03%, and 0.40±6.60%, respectively. The Pearson correlation coefficients between the ordinary and regularized NLS are 0.995 0.993, and 0.995 for $\bar{K}$, $K_{⊥}$, and $K_{\|}$, respectively. The effect of the regularized NLS estimator on the shape of the kurtosis tensor from voxels near the mid‐sagittal plane of the genus of the corpus callosum is highlighted in Figure 4B. The regularized NLS estimator shows to resolve the higher kurtosis values perpendicularly to the diffusion tensor main direction for both non‐problematic and problematic voxels.

### 3.3 Reproducibility

**FIGURE 5:**
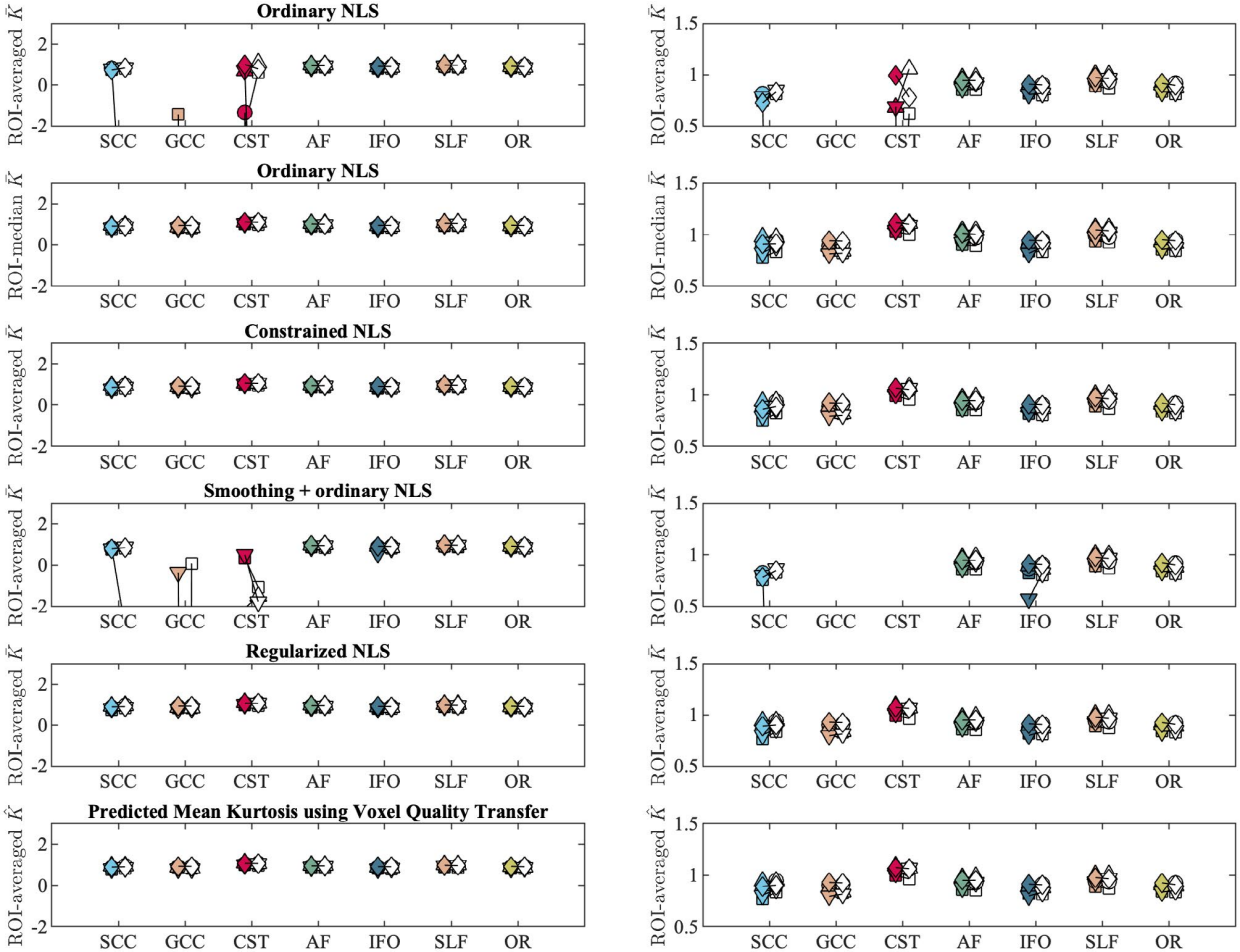
The tract‐averaged $\bar{K}$ using various fitting strategies and $\hat{K}$ (bottom row) for the test (filled marker) and retest data (open marker) each subject (labeled by marker shape). The graphs on the right column show the same data, but windowed differently for enhanced contrast. Seven major white matter tracts were evaluated: genu and splenium of the corpus callosum (GCC and SCC), corticospinal tract (CST), arcuate fasciculus (AF), inferior fronto‐occipital fasciculus (IFO), superior longitudinal fasciculus (SLF), and optic radiation (OR)

Figure 5 shows the mean $\bar{K}$ within each ROI as computed in 5 subjects using various fitting strategies for the test and retest data. In addition, we include the median $\bar{K}$ within each ROI for the ordinary NLS estimator.

In various tracts, that is, the SCC, GCC, and CST, the test‐retest reproducibility is poor when using ordinary NLS estimators, with and without spatial smoothing, because in at least one of the scans the negative kurtosis outliers dominate the ROI‐averaged $\bar{K}$. The median operator is robust to such outliers and can be used as a reference target for reproducibility analysis.

The test‐retest reproducibility is greatly improved by the use of the regularized fitting algorithm. The TRV of the ROI‐averaged metrics, computed over the 5 subjects, is tabulated per ROI in Table 2.

**TABLE 2:** The test‐retest variability (%) in the estimation of $\bar{K}$ for various tracts and fitting strategies

	GCC	SCC	CST	AF	IFO	SLF	OR
Ordinary NLS	‐	‐	‐	1.66	2.21	1.62	2.38
Median	4.20	2.19	2.17	1.93	2.60	1.68	2.03
Constrained NLS	5.30	2.89	2.16	1.75	1.75	1.59	1.99
Smoothing + ordindary NLS	‐	‐	‐	1.65	7.51	1.63	2.37
Regularized NLS	4.76	2.08	2.27	1.65	2.10	1.49	1.77
$\hat{K}$	4.73	2.08	2.28	1.65	2.10	1.49	1.76

*Notes*: If the test‐retest variability is dominated by outliers, that is, >100%, the value is not listed. To illustrate the reproducibility of the predicted mean kurtosis $\hat{K}$, we also show those results (bottom row).

The percentage of negative $\bar{K}$ estimates per tract strongly impacts the TRV. The regularized NLS estimator decimated, if not nullified, this number in comparison to the ordinary NLS estimator, with and without smoothing. Importantly, 80‐90% of the voxels with negative $\bar{K}$ using the ordinary NLS estimator converged to plausible, positive, values with regularized NLS estimator if the optimization was initiated with the outcome of the ordinary NLS estimator. For the remaining 10‐20%, the regularized fit needed to be repeated with a starting point that was obtained from the constrained NLS estimator.

### 3.4 General applicability

**FIGURE 6:**
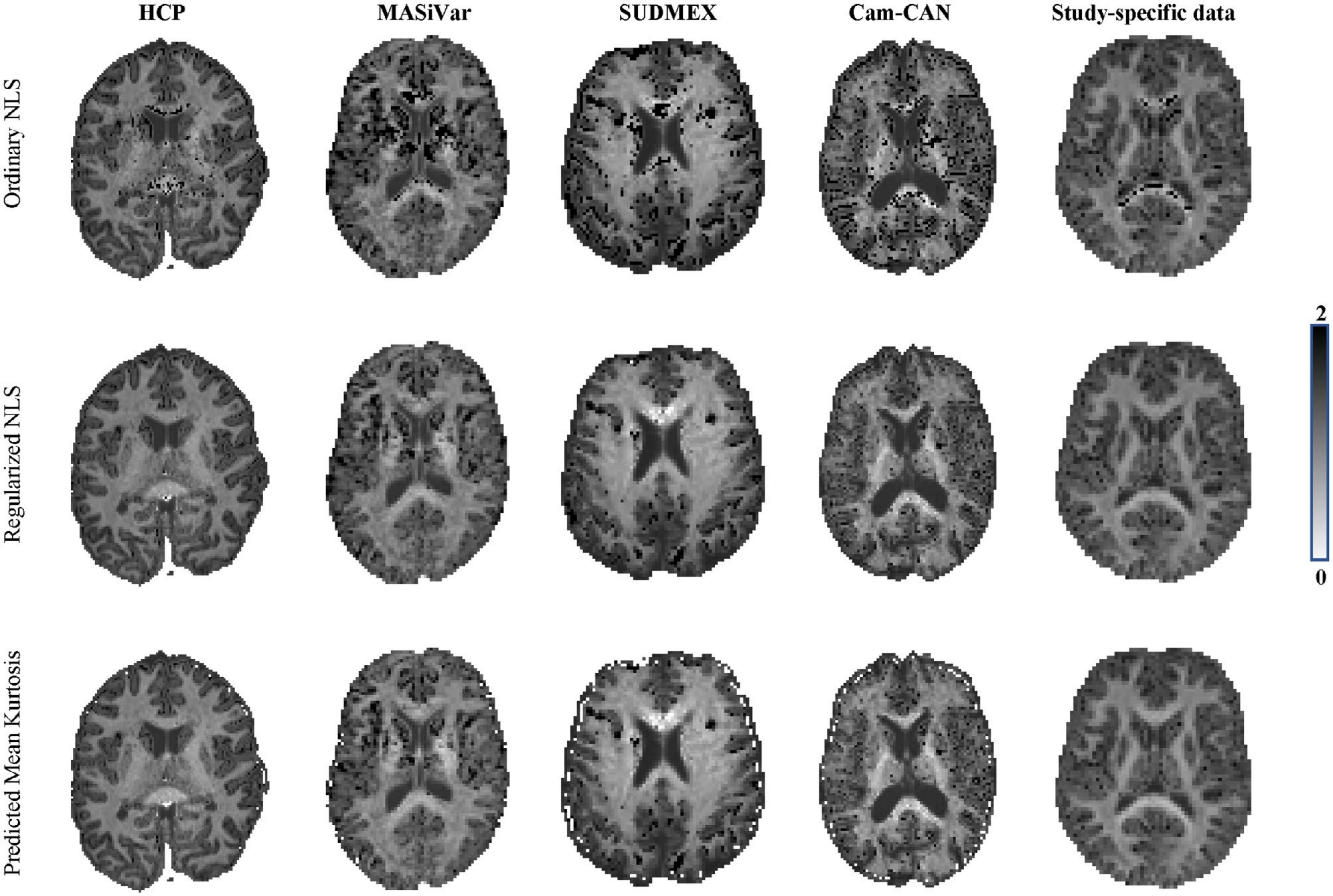
The $\bar{K}$ maps for the various dataset are shown for the ordinary and regularized NLS in the top and middle row, respectively. Moreover, we show the map of the predicted mean kurtosis $\hat{K}$ (bottom row) to illustrate the similarity in contrast

**FIGURE 7:**
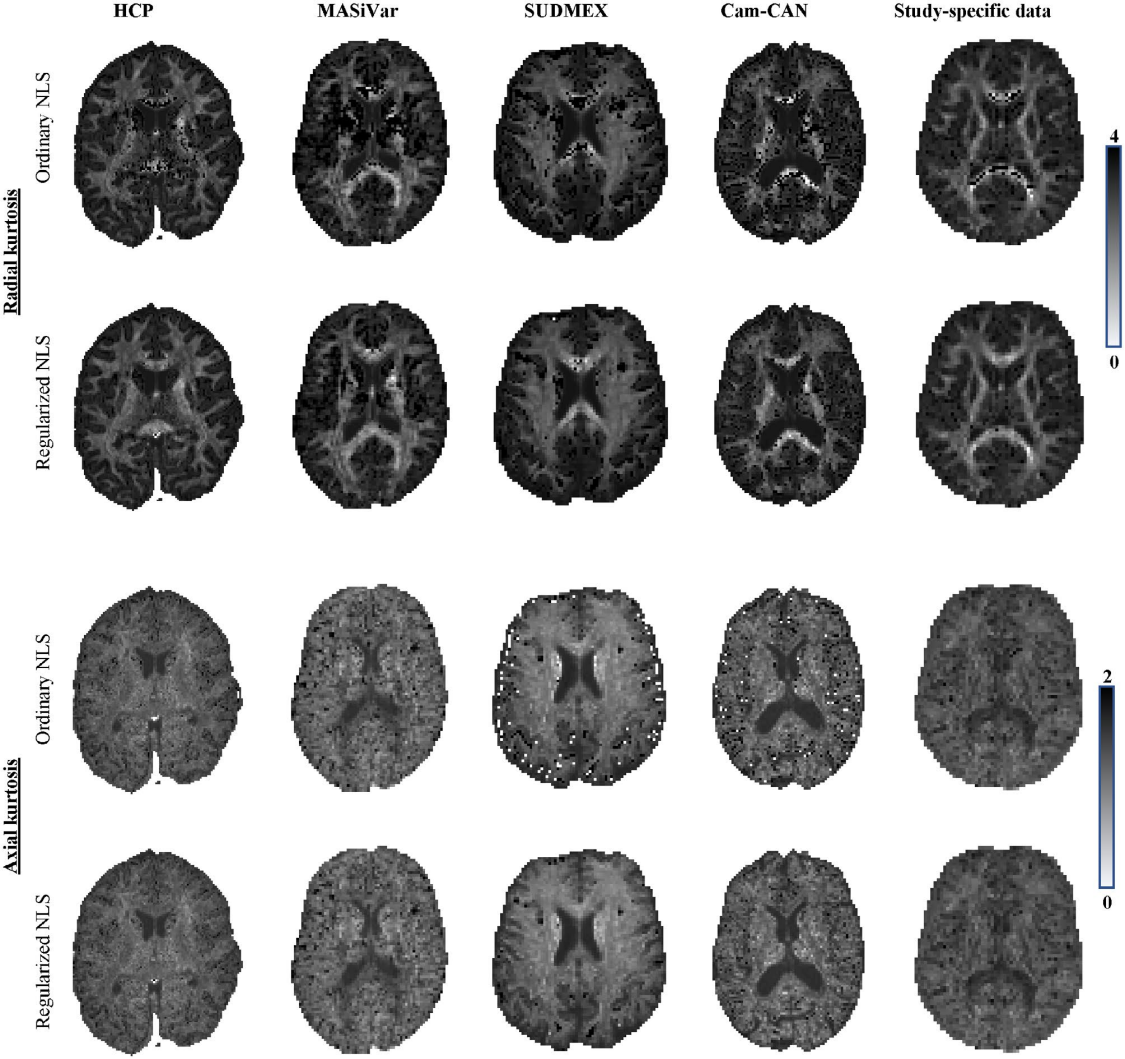
The $K_{⊥}$ and $K_{\|}$ maps for the various dataset are shown for the ordinary and regularized NLS in the top and bottom row of each panel

In Figures 6 and 7, we show the maps of $\bar{K}$, $K_{⊥}$ and $K_{\|}$ for a single, but representative slice of various data sets. The data quality varied highly from 1 dataset to another due to variations in SNR and number of diffusion‐weighted images. However, in each dataset, traditional kurtosis estimation suffered from low robustness and many negative $\bar{K}$ in structures such as the CC. Overall, the quality of the maps improved drastically for all datasets, with a few remaining black voxels (negative kurtosis values), mostly around some GM/WM boundaries, where the kurtosis and their reference values are not well understood yet.^54^ In Figure 6, we also show, for comparison, the map of the predicted mean kurtosis $\hat{K}$.

## 4 DISCUSSION

Improving the robustness of DKI fitting has been an active topic of research since the introduction of the technique. *Black voxels* have been intrinsic to DKI and challenge the visual and statistical analysis of potentially clinically relevant biomarkers of tissue integrity. Despite increasing evidence of the potential value of DKI during the past decade, the lack of a robust parameter estimation prevents widespread clinical/diagnostic adoption of the kurtosis biomarkers.

It might be impossible to attribute a single cause of the vulnerability of DKI to outliers. It has been shown that thermal noise alone can result in extremely negative kurtosis values.^49^ However, imaging artifacts, such as signal voids, Gibbs ringing, CSF pulsation, and/or misalignment of the diffusion‐weighted images might also contribute to the problem, even when the artifacts are largely corrected using state‐of‐the‐art image processing tools.^12^, ^52^, ^53^ The wide variety of signal fluctuations that might cause the DKI estimator to fail motivated the use of a strong image smoothing prior to parameter fitting and/or constraining the parameter estimation by enforcing positive kurtosis estimates.^7^, ^40^, ^50^, ^54^

The biophysically implausible kurtosis estimates have previously been associated with artifactually low non‐diffusion weighted signals. Motivated by this, Zhang et al^88^ recently proposed an approach in which the non‐diffusion‐weighted signal is altered in a selective data‐driven range. Although the approach yields visually appealing kurtosis maps, the alteration is strongly dependent on a user‐dependent tuning variable, while a selection criterion is missing.

**FIGURE 8:**
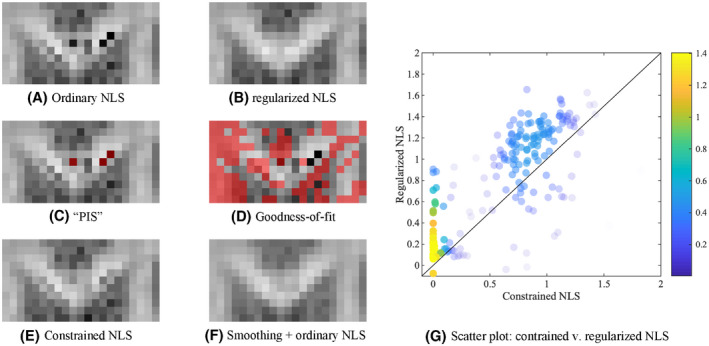
The $\bar{K}$ map of the GCC of a single subject of the reproducibility data is shown for the various fitting strategies (A, B, E, F). The grayscale intensities are scaled between 0 and 2. In (C), the voxels in which at least one diffusion‐weighted signal was larger than the non‐diffusion‐weighted signal, “physically implausible signal (PIS)” are highlighted in red. In (D), the voxels that do not satisfy the Pearson’s $\chi^{2}$ goodness‐of‐fit criterion are indicated in red. Many problematic voxels in terms of plausibility of $\bar{K}$ pass the PIS or goodness‐of‐fit criterion. G, A scatter plot shows the relationship between the $\bar{K}$ as estimated using the constrained and regularized NLS, for the voxels that were characterized with a negative $\bar{K}$ using the ordinary NLS estimator. The probability density estimate is color‐encoded

However, for >10000 voxels with negative mean kurtosis in our reproducibility data, only 18% of voxels have at least one diffusion‐weighted signal exceeding the non‐diffusion weighted signal, coined as “physically implausible signal (PIS)”^89^, ^90^; see Figure 8C. Moreover, only 63% of all such PIS voxels resulted in a negative mean kurtosis. Hence, techniques focusing on the correction of the non‐diffusion‐weighted signal might be limited to a subset of the voxels only or bias the outcomes by forcing a correction on the non‐diffusion‐weighted signal instead of considering spurious signal fluctuations across the whole of the diffusion‐weighted data.

Moreover, when comparing the fit residuals of the ordinary and regularized NLS in the voxels of interest, that is, the voxels with negative mean kurtosis, we observed that the signal prediction of the regularized NLS estimated was significantly different for each *b*‐value with both positive and negative signal offsets. This observation (data not shown) also suggests that only correcting non‐diffusion‐weighted signals is likely to be insufficient for a robust and unbiased estimator.

Up to 68% of all voxels with negative mean kurtosis within a single subject of our reproducibility data satisfied the Pearson’s $\chi^{2}$ goodness‐of‐fit criterion^91^ (Figure 8D shows in red voxels in which signals did not satisfied the Pearson’s $\chi^{2}$ goodness‐of‐fit for a zoomed brain region of the subject reproducibility data). Hence, despite the implausible kurtosis estimate, the fit residuals were normally distributed and no signal outliers were detected using a residual analysis. Robust estimators, such as RESTORE^92^ and related techniques,^93^, ^94^ would not make any difference to the outcome. Indeed, those methods are designed to improve the robustness of the fit to signal outliers, but are not necessarily tackling the problem described here.

Overall, smoothing has proven to be a very effective way to suppress the signal fluctuations that might lead to negative kurtosis.^7^, ^40^, ^50^, ^54^ However, such a brute force strategy introduces image blur and partial volume artefacts. Analogous to other artifacts, for example, Gibbs ringing,^53^, ^82^, ^90^ there is a recent trend toward the development of image processing tools that are more specifically targeted to correct a particular artifact and to avoid smoothing. The development of a robust kurtosis estimator that does not require image smoothing prior to fitting is more in line with such a strategy and, ultimately, will result in sharper images with higher effective spatial resolution.

The constrained NLS estimator only yields positive kurtosis values by design. Hence, the constrained NLS typically appears to be more accurate and robust in the estimation of DKI parameters. However, imposing constraints is likely to bias the estimator in any single voxel. In 99% of all voxels with negative $\bar{K}$ using the ordinary NLS estimator, the constrained NLS estimator provides a solution that lies on the bounds of the search landscape. Although such estimates technically satisfy the imposed constraints, they are not necessarily closer to the most biophysically plausible solution, see Figure 8.

All of these considerations are visualized and summarized in one representative figure that shows the Genu of the CC of one the study‐specific MRI data sets, Figure 8.

In this work, we demonstrated that the mean kurtosis can be very well predicted without the need to estimate the kurtosis tensor. Indeed, the powder kurtosis is already a good approximation of the mean kurtosis, despite a significant approximation error that depends on the anisotropy of the underlying process. We showed that this approximation error can be reduced significantly by using a polynomial regression model in which the mapping between the powder kurtosis, the mean diffusivity, an anisotropy index, and the mean kurtosis can be learned from the hundreds of thousands of non‐problematic voxels in the same or similar data sets. By doing so, we transfer the excellent quality of the DKI estimation in the majority of the gray and white matter voxels to the few, but persistently problematic voxels.

The accurate and robust prediction of the mean kurtosis might be relevant and sufficient for various studies. However, the regularized fitting is a necessary additional step for all studies that have interest in directional kurtosis metrics (eg, radial or axial kurtosis), DKI‐derived biophysical modeling (ie, WMTI^60^), or tractography.^63^, ^66^

Similar to the negative kurtosis, various models developed to analyze diffusion MRI data suffer from an abundance of biophysically implausible outcomes or, more subtly, a multitude of biophysically plausible solutions for which the goodness‐of‐fit is not significantly different.^95^ Although beyond the scope of this work, we hypothesize that using the regularization term presented in this work might increase the stability and precision of parameter estimators of such models. Similarly, the regularization term can be added to various variations of the loss function, including the log‐linearized or maximum likelihood function.

The implementation of this regularized estimator is publicly available on https://github.com/jelleveraart/RobustDKIFitting/.
